# Radial artery neointimal hyperplasia after transradial PCI—Serial optical coherence tomography volumetric study

**DOI:** 10.1371/journal.pone.0185404

**Published:** 2017-10-10

**Authors:** Petr Kala, Jan Kanovsky, Tereza Novakova, Roman Miklik, Otakar Bocek, Martin Poloczek, Petr Jerabek, Lenka Prymkova, Tomas Ondrus, Jiri Jarkovsky, Milan Blaha, Gary S. Mintz

**Affiliations:** 1 Department of Internal Medicine and Cardiology, University Hospital Brno and Medical Faculty of Masaryk University, Brno, Czech Republic; 2 Institute of Biostatistics and Analyses, Masaryk University, Brno, Czech Republic; 3 Cardiovascular Research Foundation, New York, New York, United States of America; Medstar Washington Hospital Center, UNITED STATES

## Abstract

**Aims:**

Transradial catheterization (TRC) is a dominant access site for coronary catheterization and percutaneous coronary interventions (PCI) in many centers. Previous studies reported higher intimal thickness of the radial artery (RA) wall in patients with a previous history of TRC. In this investigation the aim was to assess the intimal changes of RA using the optical coherence tomography (OCT) intravascular imaging in a serial manner.

**Methods and results:**

100 patients with the diagnosis of non-ST-elevation myocardial infarction (nSTEMI) treated by PCI were enrolled (6 patients were excluded from this analysis because of occluded RA at follow-up [2 patients] and insufficient quality of OCT images [4 patients]). An 54mm long OCT run of the RA was performed immediately after the index PCI and repeated 9 months later. Volumetric analyses of the intimal layer and lumen changes were conducted. Median intimal volume at baseline versus 9 months was 33.9mm^3^ (19.0; 69.4) versus 39.0mm^3^ (21.7; 72.6) (p<0.001); and median arterial lumen volume was 356.3mm^3^ (227.8; 645.3) versus 304.7mm^3^ (186.1; 582.7) (p<0.001). There was no significant difference in the effect of any clinical factor on the RA volume changes.

**Conclusions:**

OCT volumetric analyses at baseline and 9 months showed a significant increase in the radial artery intimal layer volume and a decrease in lumen volume after transradial PCI. No significant factors affecting this process were identified.

## Introduction

Many interventional cardiologists have adopted transradial catheterization (TRC) the last decade. The first TRC was performed by Campeau in 1989[1], and the first coronary stent was implanted via radial artery by Kiemeneij and Laarman in 1993[2]. Nowadays, the rate of transradial access (TRA) for percutaneous coronary interventions (PCI) is higher than transfemoral in many centers although the prevalence is higher in Asia and Europe than in the USA[3,4]. Compared to the femoral artery, TRA offers lower rate of complications such as bleeding[5] and even death[6]. However, the radial artery (RA) is smaller than the femoral artery[3], and TRA is associated with a higher prevalence of acute injuries and chronic intimal changes [7,8]. RA occlusions rate is usually low and remain mostly asymptomatic [9]. Previous studies investigated qualitative RA vessel wall changes after TRC in a non-serial manner. A greater thickness of the RA intimal layer was reported in patients with a history of TRC[8,10]. Frequency-domain optical coherence tomography (FD-OCT) uses near-infrared light for tissue imaging and has a spatial resolution close to 10 microns[11]. For RA vessel wall imaging, OCT is currently the best option to assess discreet changes of the RA wall intimal layer. Because of missing prospective data, the aim of this study was to perform first prospective serial FD-OCT study of the RA after first-time transradial PCI in consecutive patients.

## Methods

### Patient group

One hundred consecutive patients were included in the project, as a part of larger group of patients (140 subjects) enrolled into a study focusing on OCT analysis of the coronary vessels in patients with the diagnosis of myocardial infarction without ST segment elevation (nSTEMI). The following inclusion criteria were applied: diagnosis of nSTEMI, first-in-life transradial coronary catheterization, and PCI during the index procedure. Exclusion criteria included myocardial infarction with ST segment elevation (STEMI), left main coronary artery lesion, renal insufficiency with creatinine level above 150umol/l, acute heart failure, and refusal to sign the informed consent. Due to the inclusion criterion of transradial catheterization, the patients with radial artery occlusion were excluded as well. 2 patients with occluded RA at follow-up were excluded from this analysis due to unavailability of the follow-up RA OCT pullback. All patients signed written informed consent. The project was approved by the local Ethics Committee of University Hospital Brno (Brno, Czech Republic).

### Coronary angiography and PCI procedure

Cardiac catheterization was performed in accordance with the local medical standards in an 24/7 tertiary PCI center. The center has wide experience with transradial catheterizations and interventions with a 97% rate of transradial procedures in 2014. All the procedures were performed via 6F *Radiofocus Introducer II* kit (Terumo, Japan) with an intravascular sheath length of 7 cm. The RA was punctured with the kit according to local standards, using micropuncture technique with metallic entry needle and spring mini guidewire. A vasodilating drug (typically 2.5mg of verapamil) was administered in all cases, no other drugs were allowed. Solely 6F guide catheters were used for the coronary interventions. Unfractionated heparin with the target ACT ≥250 was used for the anticoagulation.

### OCT procedure protocol

**Fig 1:**
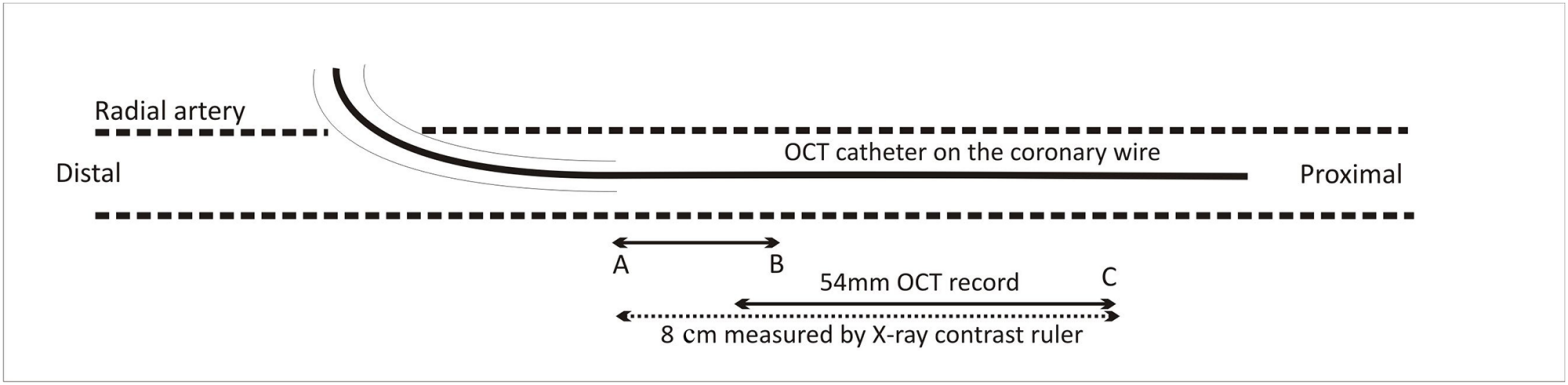
Optical coherence tomography of RA—procedure scheme. Legend: A-Sheath tip position during the OCT procedure, B-Original sheath tip position during the PCI, C-Start position of the OCT probe.

After the index coronary angiography and subsequent PCI, OCT of the RA was performed. The standard coronary wire and OCT catheter were placed in the radial artery through the 6F guiding catheter and the guiding catheter was pulled out from the radial sheath. Overall, 3cm of the sheath was withdrawn from the artery (distally), leaving 4cm inside the RA. An X-ray contrast ruler was used to identify the start of OCT imaging and pullback 8cm proximal to the actual sheath tip position. From that point, OCT pullback recording was performed using a 100% contrast fluid to flush the vessel. The standardized length of the pullback was 54mm (Fig 1). We used the *Dragonfly Duo catheter* and *Optis Ilumien OCT system* (St.Jude, Minneapolis, MN, USA) to perform the OCT procedure. An 2,5mg of verapamil through the sheath was administered before the OCT run. The puncture site was covered with a compress band for two hours to allow hemostasis after the procedure. The standard data acquisition speed was 18mm per second, getting high resolution data from the vessel in 3 seconds (for the 54mm pullback record length).

The procedure was repeated 9 months after the index procedure during follow-up coronary catheterization. The same OCT protocol as described above was used.

### OCT analysis

**Fig 2:**
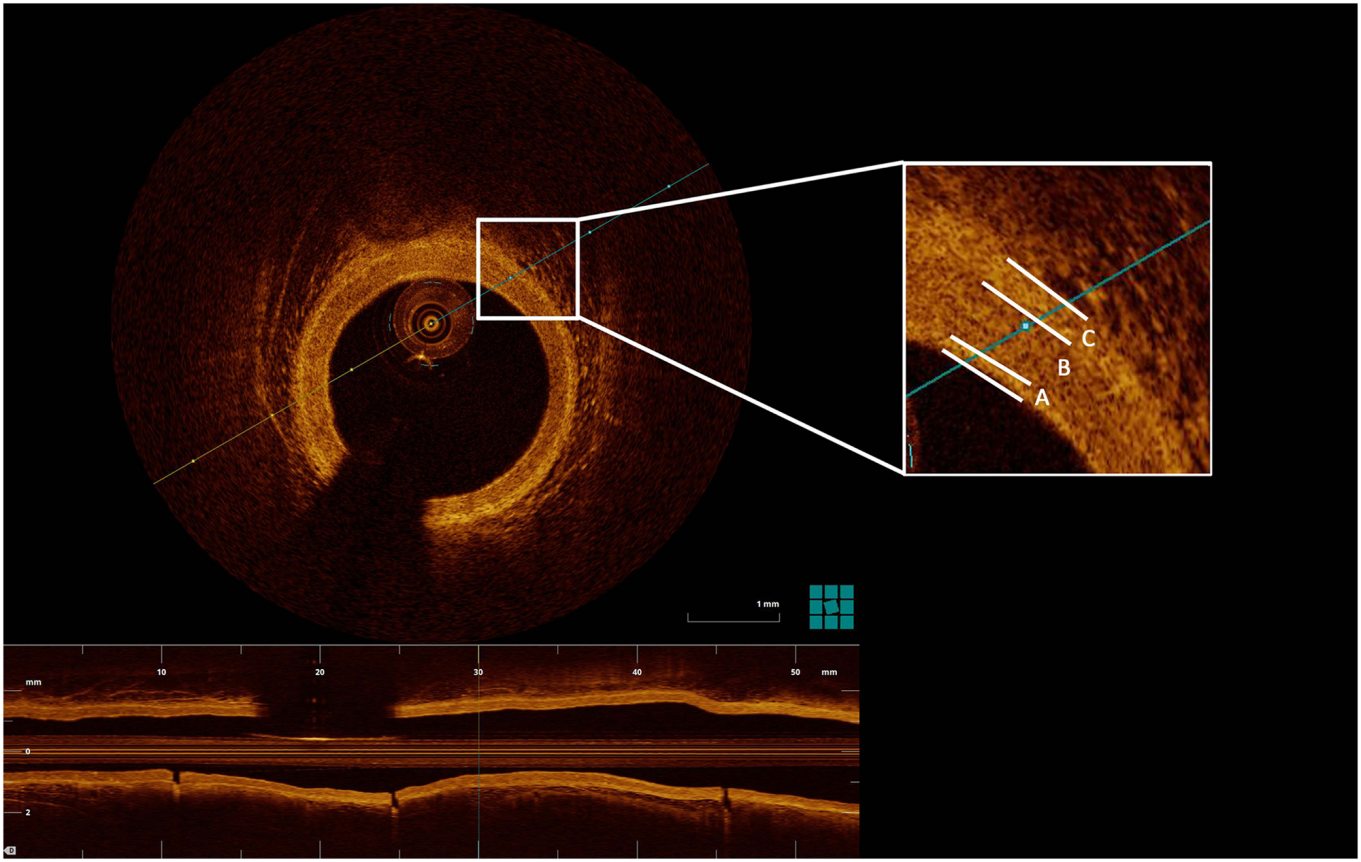
Representative OCT cross-section frame of the radial artery and its analysis. Legend: A-intimal layer, B-media, C-adventitia.

OCT images of the entire 54 mm segment for each patient were analyzed offline manually by two experienced OCT analysts (Fig 2) at baseline and follow-up. The lumen border and intima-media border was segmented every 3 mm. Simpson’s rule was applied to create a volumetric model of lumen and intimal layer of the radial artery. This model was used to compare the baseline and follow-up volumes of both the lumen and the intimal layer of the radial artery. If some frames were not of sufficient quality for evaluation, the analysis was normalized for the standard length of 54mm. The percentage of analysed frames was 94.7% both in baseline and follow-up pullbacks (Table 1). An analysis of factors affecting the volume changes was performed. In 4 patients, the quality of baseline and/or follow-up OCT was not suitable for analysis because of the insufficient blood wash-out. These patients were not included in the analysis.

**Table 1:** Evaluability of artery volumes (N = 96).

	Mean ± SD	Median (min-max)
**Baseline measurement**		
**Evaluable part of artery (%)**	**90.4 ± 12.3**	**94.7 (52.6; 100.0)**
Number of invalid frames	**1.8 ± 2.3**	**1.0 (0.0; 9.0)**
**Follow-up measurement**		
**Evaluable part of artery (%)**	**89.4 ± 13.7**	**94.7 (47.4; 100.0)**
**Number of invalid frames**	**2.0 ± 2.6**	**1.0 (0.0; 10.0)**

### Statistical analysis

Standard descriptive statistical methods were applied in the analysis; absolute and relative frequencies for categorical variables and median with 5th-95th percentile range for continuous variables. Statistical significance of differences between various subgroups of patients in baseline and follow-up measurements was tested using a non-parametric Mann-Whitney U test and Kruskal-Wallis test. Intra-individual differences in arterial volumes were assessed using a Wilcoxon signed-rank test. Influence of duration of catheterization on change of volume was tested by Mann-Whitney U test. Correlation between duration of catheterization and change of volume was tested by Pearson’s correlation coefficient. Statistical analyses were computed using SPSS 22.0.0.1 (IBM Corporation, 2014).

## Results

Radial artery OCT was well tolerated by patients with a general mild discomfort in the forearm during the contrast flush, but no clinically significant adverse events occurred.

Overall, 96 RA data records were of sufficient quality for the analysis. The median age of the group was 66.5 years. More men (67.7%) than women were enrolled. The baseline characteristics of the patient population have been listed in Table 2.

**Table 2:** Baseline characteristics.

Characteristics		N (%) or median (5^th^-95^th^ percentile)
**Gender**	Man	65 (67.7%)
	Woman	31 (32.3%)
**Age**	(N = 96)	66.5 (45.0; 80.7)
**Body-mass index**	(N = 92)	28.2 (23.1; 37.1)
**Hypertension**		65 (67.7%)
**Dyslipidemia**		31 (32.3%)
**Diabetes mellitus**		33 (34.4%)
**Peripheral vasculopathy**		4 (4.2%)
**Smoking**	Smoker	26 (27.7%)
	Former smoker	29 (30.9%)
	Never smoked	39 (41.5%)
**Alcohol**	≥ 1 drink / week	14 (14.9%)
	≥ 1 drink / month	33 (35.1%)
	< 1 drink / month	47 (50.0%)
**Creatinine (μmol/l)**	(N = 82)	87.5 (52.0; 118.0)

**Fig 3:**
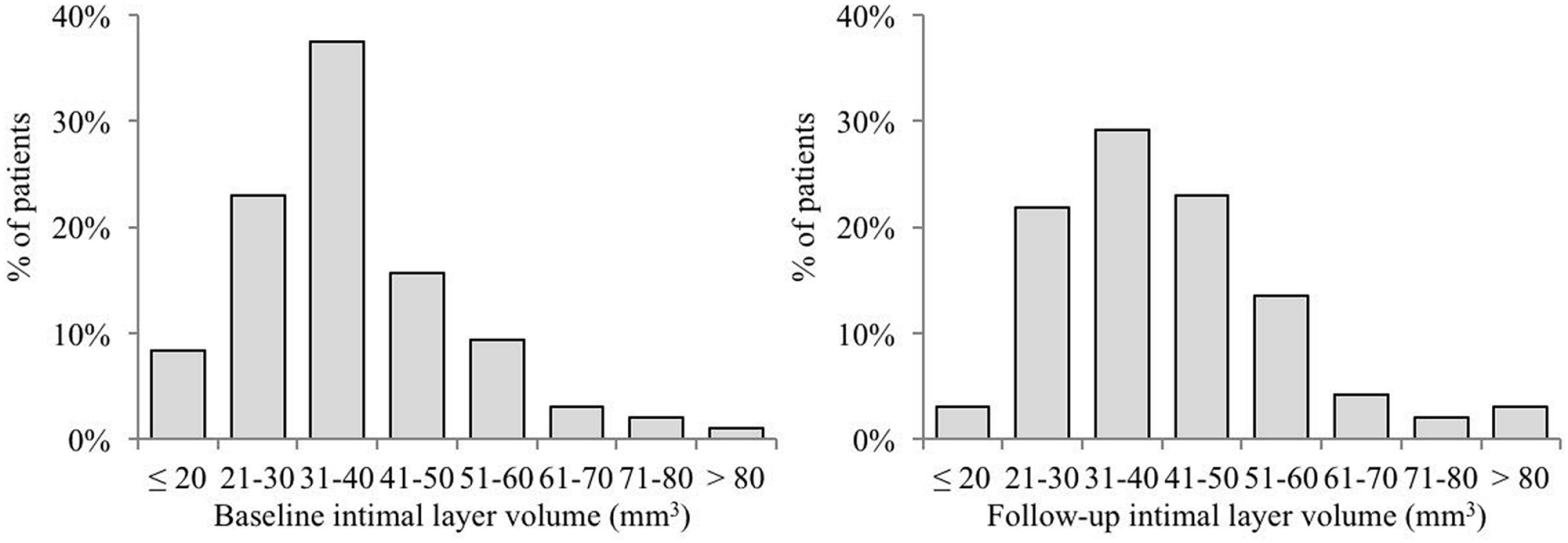
Distributions of RA intimal layer volume in baseline and follow-up measurements.

**Fig 4:**
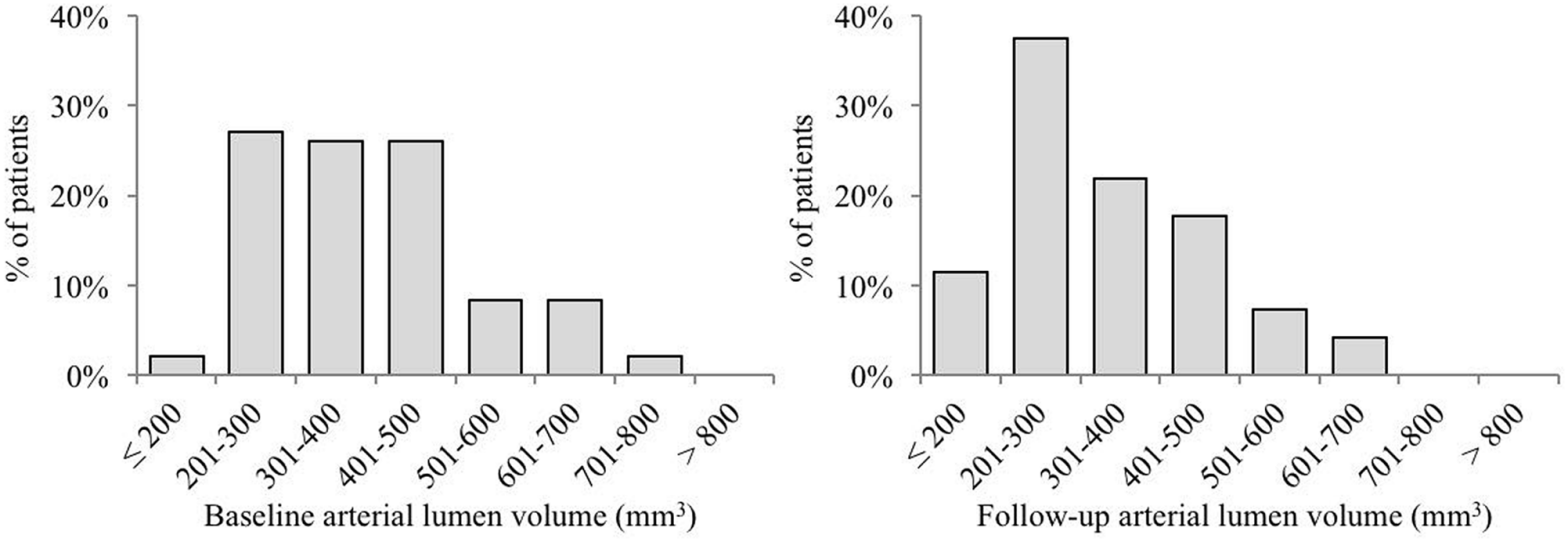
Distributions of RA luminal volume in baseline and follow-up measurements.

Irrespective of the fact that 54mm of artery was imaged in each patient, distributions of intimal layer volume were relatively wide, from 20mm^3^ to 80mm^3^ (Fig 3). Similarly, distributions of lumen volume were also wide, from 200mm^3^ to 800mm^3^ (Fig 4).

**Fig 5:**
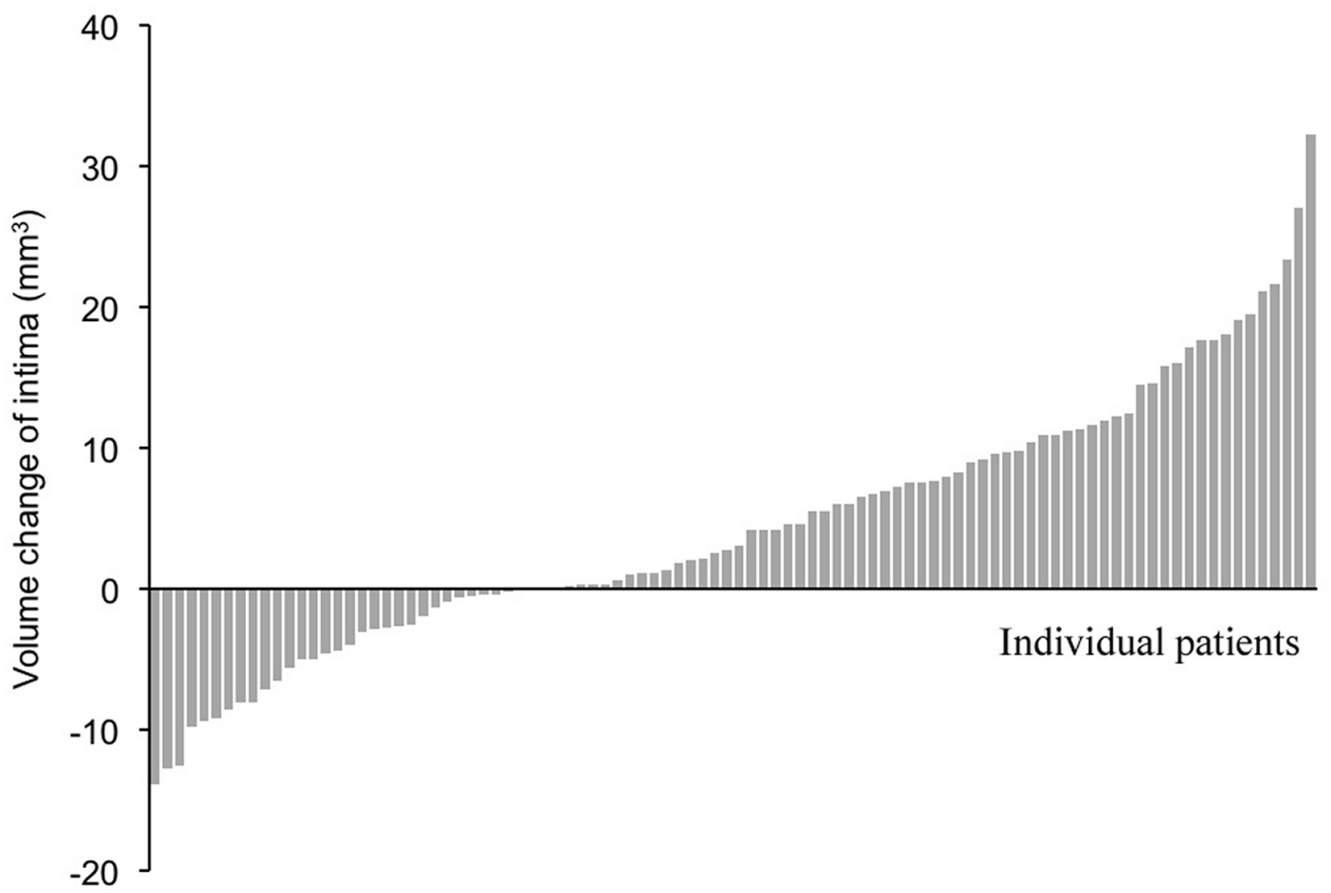
Change in the volume of intima layer of the arterial wall in individual patients (mm^3^).

**Fig 6:**
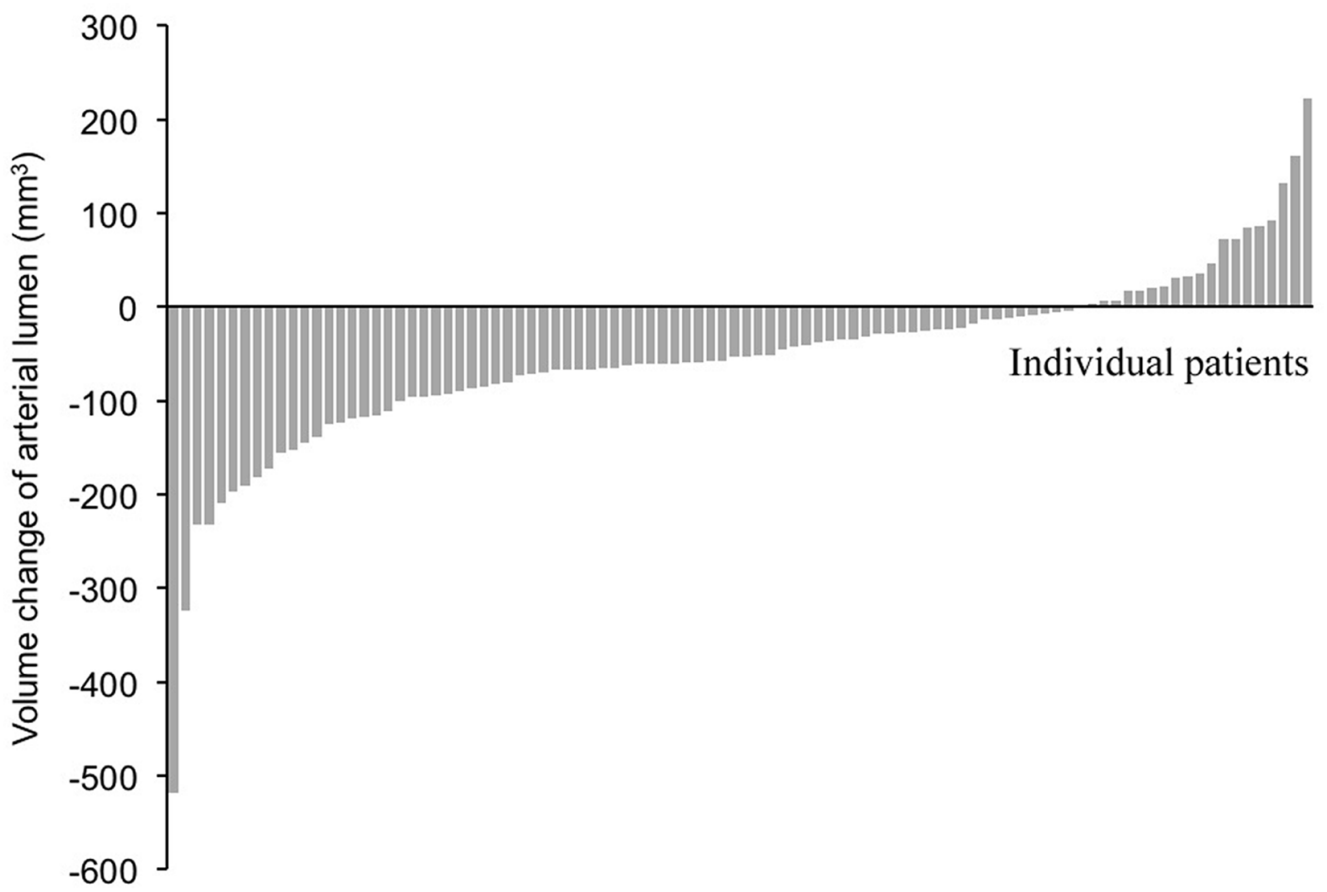
Change in the volume of arterial lumen in individual patients (mm^3^).

Median intimal layer volume at baseline was 33.9mm^3^ (19.0; 69.4) versus 39.0mm^3^ (21.7; 72.6) measured 9 months later. This difference of 3.0mm^3^ (-9.4; 21.3) was highly statistically significant (p<0.001, Table 3). The intimal volume increased in 66.7% of patients; no change or decreased volume occurred in 33.3% of patients (Table 4, Fig 5). Median lumen volume at baseline was 356.3mm^3^ (227.8; 645.3) versus 304.7mm^3^ (186.1; 582.7) 9 months later. The difference of -54.0mm^3^ (-210.6; 87.2) was highly statistically significant (p<0.001, Table 3). The lumen volume decreased in 79.2% of patients; there was no change or increased volume in 20.8% of patients (Table 4, Fig 6).

**Table 3:** RA arterial wall and lumen changes.

	N	Baseline^1^	Follow-up^1^	Difference^1^	p
**Arterial wall volume (mm**^**3**^**)**	96	33.9 (19.0; 69.4)	39.0 (21.7; 72.6)	3.0 (-9.4; 21.3)	**< 0.001**
**Arterial lumen volume (mm**^**3**^**)**	96	356.3 (227.8; 645.3)	304.7 (186.1; 582.7)	-54.0 (-210.6; 87.2)	**< 0.001**

^1^ Median (5^th^-95^th^ percentile);

**Table 4:** Change of volume (N = 96).

Volume change	Increase	Decrease
Intima layer	64 (66.7%)	32 (33.3%)
Arterial lumen	20 (20.8%)	76 (79.2%)

Analysis of multiple factors affecting intimal and lumen volume changes was performed (gender, age, body-mass index, clinical risk factors and duration of catheterization). No significant risk factor associated with the intimal and lumen volume changes was identified (Tables 5 and 6). Likewise, there was no correlation between duration of catheterization and change of volume (Tables 7 and 8).

**Table 5:** Influence of risk factors on RA arterial wall changes.

		N	Arterial wall volume		
			Baseline^1^	Follow-up^1^	Difference^1^
**Gender**	Man	65	36.5 (22.2; 69.7)	41.7 (25.5; 69.1)	4.3 (-9.8; 18.2)
	Woman	31	30.4 (15.9; 49.1)	34.5 (18.3; 72.6)	2.0 (-7.2; 27.2)
	**p**				**0.664**
**Age**	< 60	29	26.6 (16.5; 69.0)	28.6 (21.7; 60.0)	2.9 (-12.8; 19.2)
	60–69	38	35.6 (19.2; 58.4)	39.5 (19.9; 64.6)	1.8 (-9.8; 19.6)
	≥ 70	29	36.4 (22.0; 77.1)	44.4 (22.5; 84.0)	7.1 (-8.1; 27.2)
	**p**				**0.307**
**Body-mass index**	< 25	12	25.8 (15.9; 43.3)	33.0 (17.4; 59.9)	7.9 (-4.7; 23.6)
	25–29	48	36.4 (19.8; 69.7)	41.8 (24.1; 69.1)	3.7 (-8.1; 18.2)
	≥ 30	32	34.3 (19.1; 58.4)	35.0 (20.5; 75.0)	2.4 (-12.8; 27.2)
	**p**				**0.368**
**Hypertension**	Yes	65	34.7 (19.2; 69.7)	42.2 (22.7; 75.0)	4.3 (-8.7; 19.6)
	No	31	30.4 (16.5; 52.7)	31.7 (21.7; 60.0)	2.3 (-12.6; 21.8)
	**p**				**0.692**
**Dyslipidemia**	Yes	31	34.1 (22.1; 77.1)	38.7 (22.7; 84.0)	4.7 (-8.1; 19.2)
	No	65	33.7 (18.4; 59.4)	39.3 (21.7; 69.1)	2.7 (-9.8; 21.8)
	**p**				**0.848**
**Diabetes mellitus**	Yes	33	36.4 (19.0; 69.7)	43.3 (22.7; 75.0)	4.8 (-9.8; 27.2)
	No	63	33.3 (19.1; 69.0)	36.1 (21.7; 60.0)	2.2 (-9.3; 18.2)
	**p**				**0.159**
**Smoking**	Smoker	26	33.2 (18.4; 55.7)	38.2 (23.7; 63.4)	6.9 (-9.3; 19.2)
	Former smoker	29	37.1 (23.8; 77.1)	39.9 (26.7; 84.0)	2.9 (-9.4; 21.3)
	Never smoked	39	33.7 (19.0; 59.4)	38.7 (18.3; 69.1)	1.5 (-8.1; 23.6)
	**p**				**0.707**
**Alcohol**	≥ 1 drink / week	14	32.5 (12.8; 59.4)	40.7 (19.9; 63.4)	6.7 (-8.7; 21.8)
	≥ 1 drink / month	33	36.6 (19.1; 69.0)	44.4 (21.7; 75.0)	1.3 (-9.4; 21.3)
	< 1 drink / month	47	33.7 (19.0; 73.6)	37.7 (22.5; 72.6)	4.3 (-7.2; 19.6)
	**p**				**0.726**
**Creatinine**	< 100 μmol/l	63	33.8 (19.0; 59.4)	37.6 (20.5; 68.9)	2.7 (-8.1; 19.2)
	≥ 100 μmol/l	19	33.0 (16.5; 81.5)	39.9 (21.7; 88.9)	6.6 (-4.5; 17.3)
	**p**				**0.527**

^1^ Median (5^th^-95^th^ percentile);

**Table 6:** Influence of risk factors on RA lumen changes.

		N	Arterial lumen volume		
			Baseline^1^	Follow-up^1^	Difference^1^
**Gender**	Man	65	404.6 (252.8; 675.9)	321.8 (194.2; 603.8)	-61.6 (-233.2; 87.2)
	Woman	31	305.0 (203.1; 503.6)	252.3 (173.7; 445.3)	-53.1 (-192.0; 87.2)
	**p**				**0.538**
**Age**	< 60	29	353.0 (173.8; 675.9)	302.4 (198.0; 530.3)	-42.2 (-153.9; 94.0)
	60–69	38	367.3 (231.0; 640.5)	310.6 (156.0; 571.6)	-53.5 (-325.3; 133.9)
	≥ 70	29	353.6 (227.8; 645.3)	271.1 (195.0; 603.8)	-62.6 (-192.0; 8.5)
	**p**				**0.673**
**Body-mass index**	< 25	12	268.3 (212.7; 566.5)	206.3 (149.1; 498.3)	-60.8 (-126.0; 33.8)
	25–29	48	369.1 (228.4; 684.2)	317.2 (194.8; 623.2)	-33.0 (-233.2; 74.9)
	≥ 30	32	404.5 (231.0; 622.8)	300.5 (201.7; 571.6)	-68.3 (-192.0; 87.2)
	**p**				**0.123**
**Hypertension**	Yes	65	368.9 (234.5; 645.3)	314.2 (194.0; 582.7)	-58.8 (-192.0; 87.2)
	No	31	343.5 (173.8; 635.3)	270.3 (175.3; 530.3)	-43.0 (-210.6; 94.0)
	**p**				**0.922**
**Dyslipidemia**	Yes	31	369.2 (228.4; 628.1)	306.9 (175.3; 603.8)	-54.2 (-192.0; 87.2)
	No	65	353.0 (227.8; 675.9)	299.5 (194.2; 571.6)	-46.0 (-210.6; 74.9)
	**p**				**0.947**
**Diabetes mellitus**	Yes	33	369.2 (203.1; 628.1)	314.2 (194.2; 571.6)	-58.4 (-157.2; 87.2)
	No	63	353.0 (231.0; 675.9)	302.4 (175.3; 582.7)	-46.0 (-233.2; 87.2)
	**p**				**0.826**
**Smoking**	Smoker	26	372.3 (173.8; 675.9)	271.4 (194.2; 521.7)	-58.1 (-198.4; 94.0)
	Former smoker	29	404.4 (237.0; 628.1)	377.1 (173.7; 603.8)	-60.8 (-210.6; 87.2)
	Never smoked	39	337.3 (212.7; 645.3)	306.9 (175.3; 582.7)	-39.1 (-173.4; 87.2)
	**p**				**0.604**
**Alcohol**	≥ 1 drink / week	14	332.2 (145.4; 790.8)	290.0 (149.1; 521.7)	-62.4 (-519.7; 162.9)
	≥ 1 drink / month	33	391.5 (212.7; 675.9)	367.1 (208.4; 645.8)	-60.8 (-183.4; 94.0)
	< 1 drink / month	47	349.5 (228.4; 599.7)	271.1 (175.3; 582.7)	-53.1 (-198.4; 87.2)
	**p**				**0.637**
**Creatinine**	< 100 μmol/l	63	368.9 (212.7; 640.5)	307.0 (186.1; 538.3)	-53.9 (-192.0; 94.0)
	≥ 100 μmol/l	19	338.2 (227.8; 645.3)	287.7 (156.0; 672.1)	-39.1 (-325.3; 87.2)
	**p**				**0.513**

^1^ Median (5^th^-95^th^ percentile);

**Table 7:** Influence of duration of catheterization on change of volume (N = 96).

	Volume increase	Volume decrease	p
**Intima layer**			
Duration of catheterization (in minutes, median (min-max))	50.5 (23.0; 163.0)	47.5 (24.0; 108.0)	0.892
**Arterial lumen**			
Duration of catheterization (in minutes, median (min-max))	51.0 (24.0; 163.0)	48.0 (23.0; 130.0)	0.346

**Table 8:** Correlation between duration of catheterization and change of volume (N = 96).

Volume change	r	p
**Intima layer**		
Duration of catheterization (in minutes)	0.080	0.436
**Arterial lumen**		
Duration of catheterization (in minutes)	0.043	0.680

Minority proportion of patients developed opposite trend comparing to the overall result, i.e. intimal volume decrease and lumen size increase. Statistical analysis of the known risk factors showed no statistical difference between groups with different trends in intimal volume changes (Table 9).

**Table 9:** Comparison of baseline characteristics in patients with decreased and increased volume of intima (N = 96).

Characteristics		Decrease in volume (N = 32)^1^	Increase in volume (N = 64)^1^	p
**Gender**	Man	24 (75.0%)	41 (64.1%)	0.357
	Woman	8 (25.0%)	23 (35.9%)	
**Age**		62.5 (40.8; 76.9)	67.3 (49.1; 81.1)	0.113
**BMI**		28.7 (24.8; 40.1)	28.1 (23.1; 35.4)	0.523
**Hypertension**	Yes	23 (71.9%)	42 (65.6%)	0.646
	No	9 (28.1%)	22 (34.4%)	
**Dyslipidemia**	Yes	10 (31.3%)	21 (32.8%)	0.999
	No	22 (68.8%)	43 (67.2%)	
**Diabetes mellitus**	Yes	8 (25.0%)	25 (39.1%)	0.254
	No	24 (75.0%)	39 (60.9%)	
**Peripheral vasculopathy**	Yes	0 (0.0%)	4 (6.3%)	0.298
	No	32 (100.0%)	60 (93.8%)	
**Smoking**	Smoker	8 (26.7%)	18 (28.1%)	0.961
	Former smoker	10 (33.3%)	19 (29.7%)	
	Never smoked	12 (40.0%)	27 (42.2%)	
**Alcohol addiction**	≥ 1 drink / week	6 (20.0%)	8 (12.5%)	0.369
	≥ 1 drink / month	12 (40.0%)	21 (32.8%)	
	< 1 drink / month	12 (40.0%)	35 (54.7%)	
**Creatinine (μmol/l)**		85.0 (68.0; 118.0)	87.5 (51.0; 135.0)	0.601

^1^ N (%) or median (5^th^-95^th^ percentile);

## Discussion

In our study, we analysed the effect of the first-in-life TRC in 100 patients, using serial OCT analysis. The results showed significant changes of the vessel in the period of 9 months after the first catheterization. Overall intimal volume increased and lumen size decreased in 9 months, however in both analysis a minority proportion of the patients showed intimal volume decrease and lumen size increase.

Wakeyama et al. used intravascular ultrasound (IVUS) to assess 100 radial arteries for intimal-medial changes in 2002[10]. There was intima-media thickening in repeat-TRI patients compared to the first-time TRI patients, especially in the distal radial artery. In 2008, Burris et al. used OCT for graft quality evaluation of the cadaverous radial artery after endoscopic and open harvesting[12]. The first OCT study investigating RA changes in vivo was conducted by Yonetsu et al. in 2010[8], enrolling 69 patients, dividing them into first-time and repeat-TRC groups. By measuring multiple cross-section areas of the RA, they found intimal areas to be significantly greater in the repeat-TRC RA group. Older time-domain OCT technology (TD-OCT) was used together with longer (16cm) sheath introduction.

In our study, we enrolled solely “TRC-naive” patients. Our results proved previously suggested hypothesis that even uncomplicated and relatively short TRC affects the radial artery as a complex part of the arterial vascular system. Recent publication by Nakata et al.[13] proved that 6F sheath insertion into the RA impaired vascular endothelial function the day after the procedure. The impaired changes assessed by reactive hyperemia peripheral arterial tonometry lasted for 6 months. Taken together, Taken together, these results suggest that most of the diagnostic and therapeutic catheterization are associated with negative RA changes during follow-up.

Question remains, what distinguishes the patients with the opposite trend in development, i.e. patients’ minority with intimal volume decrease and lumen volume increase. Since we have not found any differences in the risk factor analysis, we can only speculate on the reasons. We could blame unknown genetic factors, operator variability in catheter manipulation or even unmeasured variables like the degree of antiplatelet therapy.

Due to the fact that no other factors have proved to have a strong effect on the radial artery changes, it may be observed that the RA was affected solely by TRC. Recently, a comprehensive review on minimizing RA damage has been published[14].

However, in the real-life setting, rather in daily practice, there are numerous and heterogenous factors that can impact RA degree of injury, chronic changes or even patency: different amount of heparin administered in different centers, number of previous transradial catheterization, hydrophilic sheaths, degree of spasm, size of the catheter etc.

### Limitations

The analysis was limited to 54mm, and the OCT was performed only at baseline and 9 month follow-up; therefore, we could not assess the true time-course of post-TRC changes. There are numerous specific variables that could not be controlled, such as degree of catheter manipulation, operator interpersonal variability, number of catheter exchanges etc. However, we showed no correlation of the results with the duration of the catheterization.

## Conclusion

The volumetric model of the radial artery lumen and the arterial wall intimal layer after transradial PCI assessed by OCT at baseline and at 9-month follow-up showed a significant effect of transradial catheterization. The intimal layer volume increased significantly, while the volume of the lumen decreased. No significant clinical factors affecting this process have been found.

## References

[1] Campeau L. Percutaneous radial artery approach for coronary angiography. Cathet Cardiovasc Diagn. 1989 1;16(1):3–7. 2912567 10.1002/ccd.1810160103

[2] Kiemeneij F, Laarman GJ. Percutaneous transradial artery approach for coronary stent implantation. Cathet Cardiovasc Diagn. 1993 10;30(2):173–8. 8221875 10.1002/ccd.1810300220

[3] Caputo RP, Tremmel JA, Rao S, Gilchrist IC, Pyne C, Pancholy S, et al Transradial arterial access for coronary and peripheral procedures: executive summary by the Transradial Committee of the SCAI. Catheter Cardiovasc Interv Off J Soc Card Angiogr Interv. 2011 11 15;78(6):823–39. 10.1002/ccd.23052 21544927

[4] Bertrand OF, Rao SV, Pancholy S, Jolly SS, Rodés-Cabau J, Larose E, et al Transradial approach for coronary angiography and interventions: results of the first international transradial practice survey. JACC Cardiovasc Interv. 2010 10;3(10):1022–31. doi: 10.1016/j.jcin.2010.07.013 20965460 10.1016/j.jcin.2010.07.013

[5] Jolly SS, Yusuf S, Cairns J, Niemelä K, Xavier D, Widimsky P, et al Radial versus femoral access for coronary angiography and intervention in patients with acute coronary syndromes (RIVAL): a randomised, parallel group, multicentre trial. Lancet. 2011 4 23;377(9775):1409–20. doi: 10.1016/S0140-6736(11)60404-2 21470671 10.1016/S0140-6736(11)60404-2

[6] Chase AJ, Fretz EB, Warburton WP, Klinke WP, Carere RG, Pi D, et al Association of the arterial access site at angioplasty with transfusion and mortality: the M.O.R.T.A.L study (Mortality benefit Of Reduced Transfusion after percutaneous coronary intervention via the Arm or Leg). Heart Br Card Soc. 2008 8;94(8):1019–25. 10.1136/hrt.2007.136390 18332059

[7] Novakova T, Kanovsky J, Miklik R, Bocek O, Poloczek M, Jerabek P, et al Short sheath benefit in radial artery injury after PCI—optical coherence tomography serial study. Biomed Pap Med Fac Univ Palacky Olomouc Czechoslov. 2016 9;160(3):393–8. 10.5507/bp.2016.035 27641357

[8] Yonetsu T, Kakuta T, Lee T, Takayama K, Kakita K, Iwamoto T, et al Assessment of acute injuries and chronic intimal thickening of the radial artery after transradial coronary intervention by optical coherence tomography. Eur Heart J. 2010 7;31(13):1608–15. doi: 10.1093/eurheartj/ehq102 20413398 10.1093/eurheartj/ehq102

[9] Mattea V, Salomon C, Menck N, Lauten P, Malur FM, Schade A, et al Low rate of access site complications after transradial coronary catheterization: A prospective ultrasound study. Int J Cardiol Heart Vasc. 2017 3;14:46–52. doi: 10.1016/j.ijcha.2016.12.003 28616563 10.1016/j.ijcha.2016.12.003 PMC5454178

[10] Wakeyama T, Ogawa H, Iida H, Takaki A, Iwami T, Mochizuki M, et al Intima-media thickening of the radial artery after transradial intervention: An intravascular ultrasound study. J Am Coll Cardiol. 2003 4 2;41(7):1109–14. 12679209 10.1016/s0735-1097(03)00089-5

[11] Bezerra HG, Costa MA, Guagliumi G, Rollins AM, Simon DI. Intracoronary optical coherence tomography: a comprehensive review clinical and research applications. JACC Cardiovasc Interv. 2009 11;2(11):1035–46. doi: 10.1016/j.jcin.2009.06.019 19926041 10.1016/j.jcin.2009.06.019 PMC4113036

[12] Burris NS, Brown EN, Grant M, Kon ZN, Gibber M, Gu J, et al Optical coherence tomography imaging as a quality assurance tool for evaluating endoscopic harvest of the radial artery. Ann Thorac Surg. 2008 4;85(4):1271–7. doi: 10.1016/j.athoracsur.2007.12.044 18355508 10.1016/j.athoracsur.2007.12.044 PMC2636971

[13] Nakata T, Ikeda S, Koga S, Yoshida T, Koide Y, Kawano H, et al Impact of Catheter Sheath Insertion into the Radial Artery on Vascular Endothelial Function Assessed by Reactive Hyperemia Peripheral Arterial Tonometry. Int Heart J. 2015;56(5):489–94. doi: 10.1536/ihj.15-094 26370365 10.1536/ihj.15-094

[14] Mamas MA, Fraser DG, Ratib K, Fath-Ordoubadi F, El-Omar M, Nolan J, et al Minimising radial injury: prevention is better than cure. EuroIntervention J Eur Collab Work Group Interv Cardiol Eur Soc Cardiol. 2014 11;10(7):824–32. 10.4244/EIJV10I7A142 24472679

